# What do patients really know? An evaluation of patients’ physical activity guideline knowledge within general practice^1^

**DOI:** 10.1080/17571472.2016.1173939

**Published:** 2016-05-25

**Authors:** S. Morton, D. Thompson, P. Wheeler, G. Easton, A. Majeed

**Affiliations:** ^a^ Department of Primary Care and Public Health, School of Public Health, Imperial College London, London, UK; ^b^ Department for Sport & Exercise Medicine, University Hospitals of Leicester NHS Trust, Leicester, UK; ^c^ School for Sport, Exercise, & Health Sciences, Loughborough University, Loughborough, UK; ^d^ National Centre for Sport & Exercise Medicine – East Midlands (NCSEM-EM), Loughborough, UK

**Keywords:** National physical activity guidelines, general practice, exercise interventions, physical activity

## Abstract

**Background:**

Physical inactivity is well recognised as one of the leading causes of preventable death. However, little is known about the general public’s knowledge surrounding national physical activity guidelines, particularly within general practice (GP).

**Setting:**

Two GPs (York and Maidenhead, UK).

**Question:**

Are GP patients aware of the national physical guidelines? Also, are health care professionals routinely raising the issue of physical inactivity and would patients welcome support from health care professionals regarding inactivity?

**Methodology:**

A questionnaire was distributed in two GPs over a one-week period to evaluate patients knowledge of the national physical activity guidelines.

**Results:**

Ninety-four participants completed the questionnaire over one week (60 female; 34 male), with an average age of 54.2 (standard deviation: 19.9 years). 14% (95% Confidence Interval (CI): 8–22%) of the total participants correctly knew the recommended national guidelines for physical activity. 52% (95% CI: 42–63%) recalled being asked by a health care professional about their activity levels. 46% (95% CI: 35–56%) would welcome support from a health care professional around improving their activity levels.

**Discussion/Conclusion:**

Only 14% of responders correctly knew the current national minimum activity guidelines. Encouragingly 46% of participants in our study were interested in physical activity advice from a health care professional. Health care professionals need to be aware that many patients do not know the current physical activity guidelines and recognise that primary care may be an underutilised opportunity to educate and promote physical activity.

## Why this matters to me?

We feel that promotion of physical activity has the potential to positively impact the health and well-being of our patients and the general public. After arranging an undergraduate medical student conference relating to exercise medicine, we realised that virtually none of the attendees knew the national physical activity guidelines. We therefore were curious as to what knowledge the general population had relating to exercise guidelines. We were looking for ways to teach medical students how to prescribe exercise, but were unclear if our patients wanted this and whether exercise was routinely already being discussed in the community. We therefore decided to conduct this survey within GP to establish the answer to our questions.

## Key message

• The majority of the GP population are unaware of the national physical activity guidelines, despite inactivity being one of the leading preventable causes of death.
• Encouragingly around 50% of patients attending GP would like additional support from a health care professional relating to physical activity.
• Appointments in primary care are likely to be an underutilised opportunity to educate and promote physical activity in our patients.
• As a profession general practitioners should ensure they are aware of the national physical activity guidelines and impart this knowledge to patients, along with advice on how to become more physically active.

## Introduction

The impact of physical activity on health and well-being is widely documented within the scientific literature. Physical inactivity is the fourth leading cause of preventable death from non-communicable disease worldwide and was thought to contribute to 5.3 million preventable deaths in 2008 alone.[1,2] Despite this, the numbers meeting recommended guidelines for physical activity remain low; 39% of European adults are reported as never undertaking regular physical activity in their routine week.[3] In 2013, The World Health Assembly identified tackling physical inactivity as one of its priority objectives in the fight against non-communicable disease.[4,5]

An online study using Health Survey for England Data performed in 2013 found that only 18% of UK adults sampled knew the physical activity guidelines.[6] Within one workplace only 15% of adults accurately reported the guidelines, with those who had employer support to achieve greater physical activity levels being more likely to be correctly aware of the guidelines.[7] To the best of our knowledge, there is minimal literature relating to the knowledge of physical activity guidelines of patients within a general practice (GP) setting. The aim of this study was to evaluate whether patients in GP were aware of the national physical activity guidelines, as detailed in Table 1. Additionally, information was obtained regarding whether health care professionals were routinely raising the issue of physical inactivity and whether patients would welcome support from health care professionals in regards to activity levels.

**Table 1.** National guidelines for physical activity.[17]

At least 150 minutes of moderate-intensity aerobic activity such as cycling or fast walking every week
OR
75 minutes of vigorous-intensity aerobic activity such as running every week
OR
An equivalent mix of moderate- and vigorous-intensity aerobic activity every week
AND
Muscle-strengthening activities on two or more days a week that work all major muscle groups

## Methods

A single A4 page questionnaire was designed and distributed to two GPs in the United Kingdom, Practice A (York area) and Practice B (Maidenhead area). At the time of the study an author was placed at each practice for a one-week period as part of their medical degree (random selection process by the university). This random allocation of practices by the university, allowed a North vs South comparison with (according to the Census 2011), the Windsor and Maidenhead borough being ranked over 100 places higher than York in the ‘Higher managerial, administrative and professional occupations’ rankings.[8] The questionnaire included two questions for basic demographic data (age and gender) and five questions relating to – the national recommended guidelines, the amount of time patients spent doing physical activity and whether a health care professional had ever asked them about their physical activity levels (see Appendix 1). Along with a yes/no question for whether the participant knew the physical activity guidelines, there was a white space question for those who answered yes to record what they believed the guidelines to be; this is similar to the question used in the study by Knox et al.[7]

The questionnaire was distributed by the receptionist at Practice A and by the author at Practice B. There was a box at Practice A where completed questionnaires could be placed during the one-week period and at Practice B questionnaires were handed out and collected in person, also over a one-week period. Both practices had their data collected in the winter period from December to January. Practice A had the data collected in December 2013 and Practice B in January 2014 to minimise change from seasonal variation.

### Ethics

As this was an anonymous survey, which did not collect any patient-identifiable information and made no change to treatment, this project was assessed to not require formal ethics approval, based on the NHS Health Research Authority Toolkit, and instead fell under the category of service evaluation (Appendix 2).[9] It received appropriate internal permissions from within the two GP surgeries, and patients were informed that they were able to opt in or out of completing the questionnaire without it influencing their health care. Consent was presumed by completion of the questionnaire.

### Data and statistical analysis

All data were recorded in Excel 2010, Windows 8 and was handled in keeping with Information Governance requirements. Data were analysed using SPSS (SPSS Statistics version 20, IBM). The chi-square test and independent t test were used to compare practices. Statistical significance was set at *p* = 0.05.

## Results

In total, 94 participants completed the questionnaire, 45 from Practice A and 49 from Practice B. Sixty were female (29 Practice A; 31 Practice B) and 34 were male (16 Practice A; 18 Practice B). The average age was 54.2 (standard deviation (SD): 19.9 years) (58.5 years (SD: 18.7) Practice A; 50.2 years (SD: 18.5) Practice B), with a range of 16–90 years.

### Main outcome

**Figure 1.**
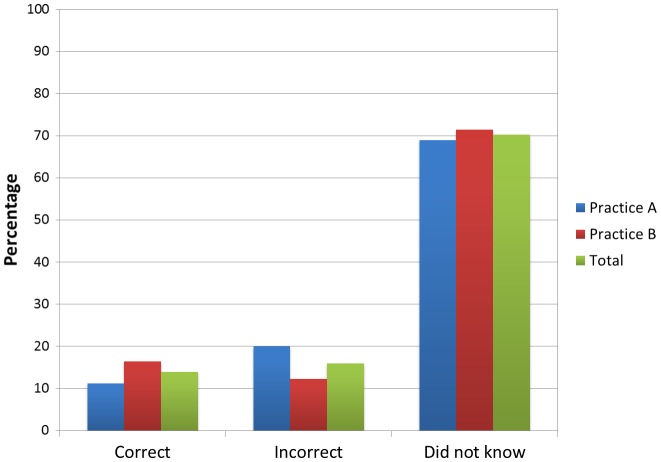
Percentage of subjects who correctly knew the national guidelines for physical activity levels, the percentage who were incorrect and the percentage who did not know what the guidelines where, for Practice A and B and the combined total.

A minority of 14% (*n* = 13, 95% confidence interval (CI): 8–22%) of the total participants knew correctly the national guidelines for physical activity levels. An additional 16% (*n* = 15, 95% CI: 9–25%) answered ‘yes’ to knowing the national guidelines but upon recording what they believed the guidelines to be, were in fact incorrect. Of the above 16%, the vast majority [87% (*n* = 13, 95% CI: 60–98%)] under-estimated the weekly exercise level guidelines. Seventy per cent of respondents (*n* = 66, 95% CI: 60–79%) stated they did not know what the guidelines were. Figure 1 details the percentage of participants in each category for each practice with comparison to the total. There was no significant difference between the practices (*χ* = 1.37, *p* = 0.51).

### Secondary outcomes

**Figure 2.**
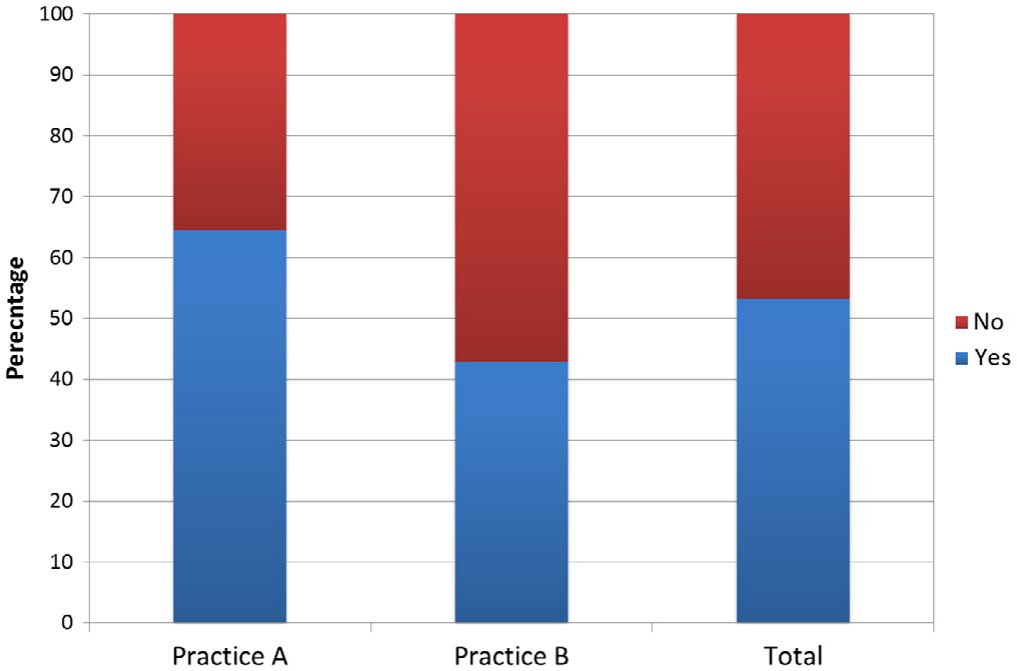
Percentage of subjects who had previously been asked by a health care professional about their physical activity levels.

Figure 2 shows the percentage of participants who recalled that they have previously been asked by a health care professional in their GP about their levels of physical activity. There was a statistically significant difference between the percentage who had been asked about their physical activity levels in the two practices, with more being asked in Practice A than Practice B (*χ* = 4.40, *p* = 0.04).

**Figure 3.**
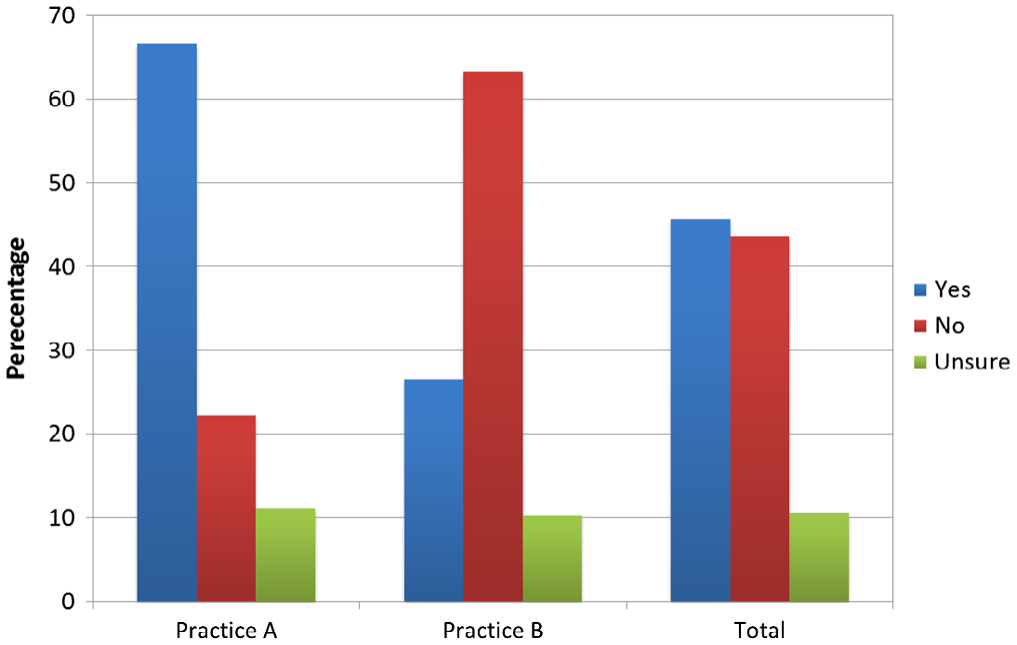
Percentage of subjects who would like more help to become physically active.

Figure 3 shows the percentage of participants who would like more help regarding their physical activity levels from their General Practitioner or the National Health Service. There was no statistically significant difference between the practices (*χ* = 0.22, *p* = 0.64).

Appendix 3 shows further analysis of the results.

## Discussion

### Main findings of this study

Our study showed that only 14% of participants knew the national guidelines for physical activity, with 13% underestimating and 2% overestimating. Also we found that just over 50% of participants had previously been asked about their physical activity levels by a health care professional. Encouragingly, 46% say that they would welcome help to become more physically active. Overall, as a snapshot of two GPs, this study provides us some insight into patient’s knowledge and attitudes towards physical activity within a GP setting, and allows us to consider opportunities to investigate this further.

### What is already known on this topic?

Our study showed that only 14% (95% CI: 8–22%) of participants correctly knew the activity guidelines, which is similar to previous studies.[6,7,10,11] Knox et al. [7] found that 8.9% underestimated and 13.8% overestimated the guidelines, respectively, in comparison to the 13% of participants who underestimated in our study and 2% who overestimated. It is also important to note that we have less information in our study relating to education levels of our participants compared to the other studies and it is likely that this is an important influence on the results.[7,11] The average age in this study was also higher than in the Knox et al. [7] study (54.2 v 38.9 years), although this is likely to represent more accurately the average age of people attending GP.

It is hardly surprising that the general population are unaware of physical activity guidelines, when research suggests that health care practitioners also are not.[12] In 2006, a study demonstrated that as few as 13% of doctors knew the current recommendations.[12] Weiler et al. [13] recently reported only 56% of UK medical schools teach the current guidance on physical activity, and it remains unclear as to how this translates into knowledge.

We found that 53% of patients had previously been asked about their level of physical activity by a health care professional, a number slightly higher than the 46% reported in the 2008 Darzi report.[14] There was a statistically significant difference between the practices, with 43% being asked in Maidenhead in comparison to 64% in York. This difference may suggest that there is inter practice variation in the approach for addressing physical inactivity. Whilst discussion of physical activity was not confirmed by reviewing GP records, this information at least shows us those discussions that were recalled by the patient. Although this would require further validation, it is likely the events that patients recall have the most potential to result in a change of behaviour. Although not a formal outcome of this study, of note was that from the participants who had not previously been asked about activity levels, 50% self-reported not meeting the national physical activity level guidelines, whereas, in the overall sample, only 28% of participants self-reported not meeting the guidelines. Knox et al. [7] found that those who correctly knew the activity-guidelines were more likely to have received employer support regarding activity levels. It may be that discussing physical activity and/or providing a form of intervention is in fact successful in altering patients’ activity levels, although more research is required to draw definitive conclusions about this.

Of the patients surveyed, 46% stated that they would be interested in receiving more help from a health care professional regarding their exercise levels. The Health Survey for England performed in 2007 found that one in four patients believed they would be more active if advised to by their GP or nurse.[15] Williams et al. [16] performed a systematic review that found promotion of physical activity in a primary care setting, for example, with motivational interviewing often including written material, had a sustained effect lasting greater than 12 months on self-reported activity versus no intervention. They found that for a clinically meaningful benefit the number needed to treat was 12 and the intervention typically was small and inexpensive. In contrast the number needed to treat for smoking cessation is reported as between 50 and 120.[2]

### Future research and training

Without a prominent public health campaign, such as ‘5-a-day’, and the lack of knowledge within the health care profession itself, it is perhaps unsurprising that the majority of the general population are unaware of their physical activity targets. A larger study within multiple GPs across the country would be beneficial to confirm these results and to establish variation across the UK and internationally. As discussed above, within medical schools and the health care profession it appears there is an urgent need for improved physical activity teaching. In the short term, further training of current HCPs on national guidelines and benefits of physical activity may enable practitioners to confidently broach this subject with their patients.

### Limitations of this study

Participants within our study essentially self-selected by volunteering to complete the questionnaire whilst at the GP. This process of recruitment means a response rate is difficult to calculate and there is likely to be an element of ascertainment bias. Also, this study is based within the United Kingdom, thereby limiting the generalisability of the research. In the future, structured interviews may be a useful tool to increase our understanding of patient’s knowledge, interpretation and learning around physical activity levels.

## Conclusions

The national guidelines for physical activity were not well known by the patients within the GP setting surveyed and almost half of patients had not previously been asked about their activity levels. Encouragingly, 46% of participants in our study were interested in physical activity advice from a health care professional. Further studies are needed to determine the most appropriate intervention for the education of health care providers and subsequently patients in a primary care setting regarding activity guidelines. The results of our study would suggest there is at present an underutilised yet significant opportunity to improve the health of patients in these areas by promotion of physical activity and by increasing the knowledge of patients around national guidelines.

## Conflict of Interests

The authors declare that there is no conflict of interest.

## Funding

This research received no specific grant from any funding agency in the public, commercial or not-for-profit sectors.

## Acknowledgements

Thanks go to the staff and patients at both the GP in York and Maidenhead.

## Governance

The survey was overseen by the general practitioner in charge at both the York and Maidenhead study.

## Figures and Tables

**Figure A1.**
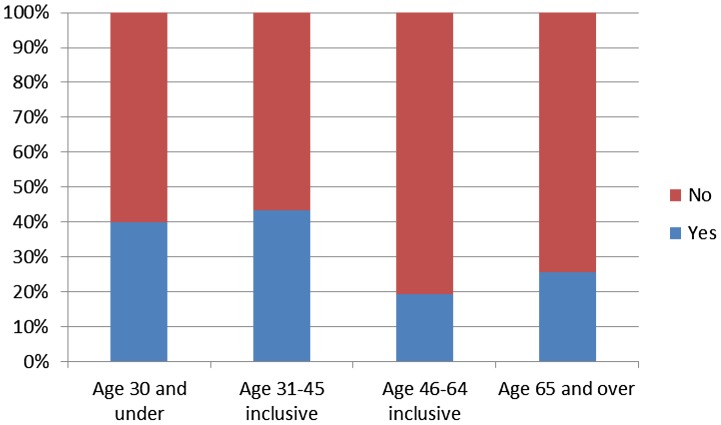
Percentage of patients who correctly knew the national physical activity guidelines divided into age groups.

**Figure A2.**
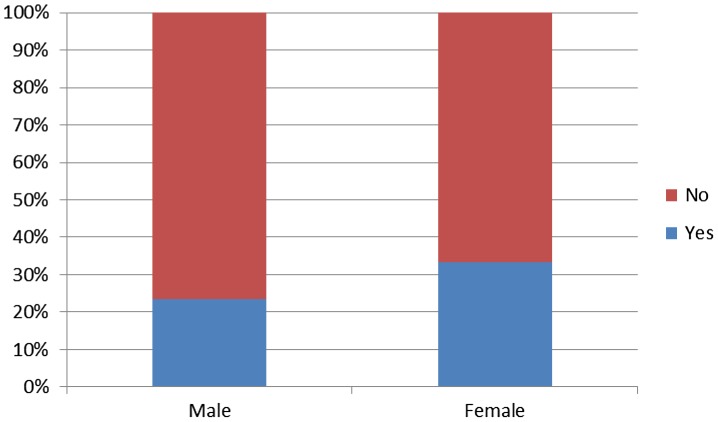
Percentage of patients who correctly knew the national physical activity guidelines divided into gender.

**Figure A3.**
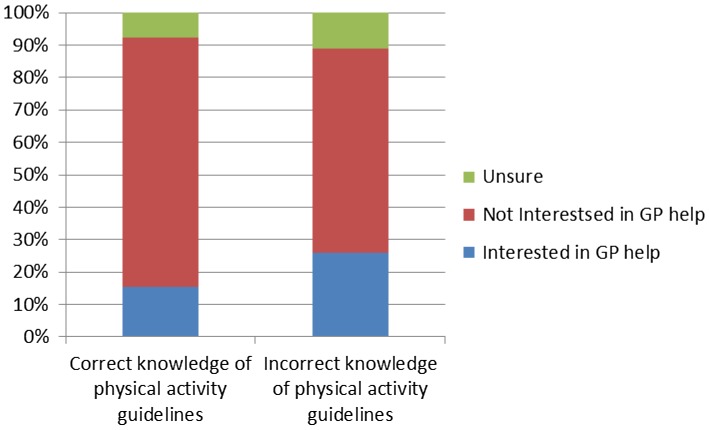
Percentage of patients who correctly knew the national guidelines and were interested in GP help in physical activity.

**Figure A4.**
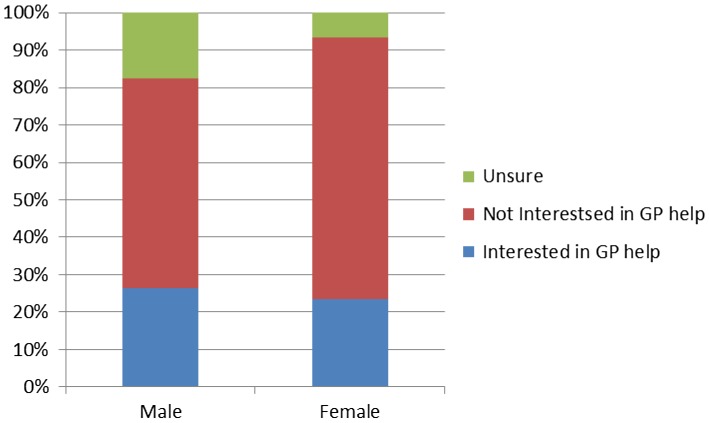
Percentage of patients who were interested in GP helps in physical activity divided by gender.

**Figure A5.**
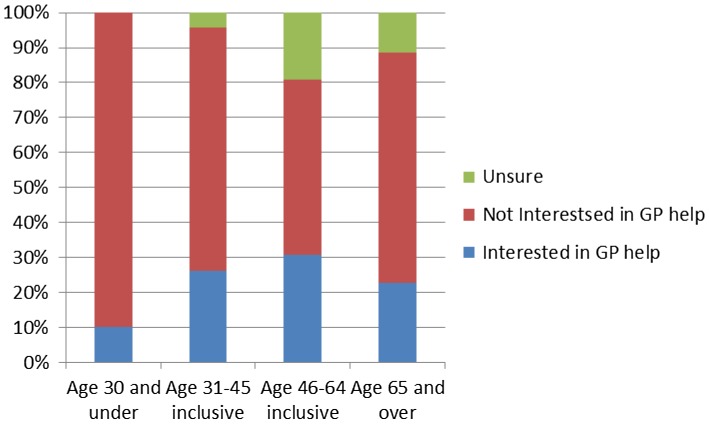
Percentage of patients who were interested in GP help divided into age groups.

## Appendix 3

^Figure A1, Figure A2, Figure A3, Figure A4, Figure A5^
